# Supplementary figures and images for: Therapeutic potential of IL6R blockade for the treatment of sepsis and sepsis-related death: A Mendelian randomisation study
Source: PLoS Med. 2023 Jan 30;20(1):e1004174. doi: 10.1371/journal.pmed.1004174 (PMC9925069; doi:10.1371/journal.pmed.1004174)

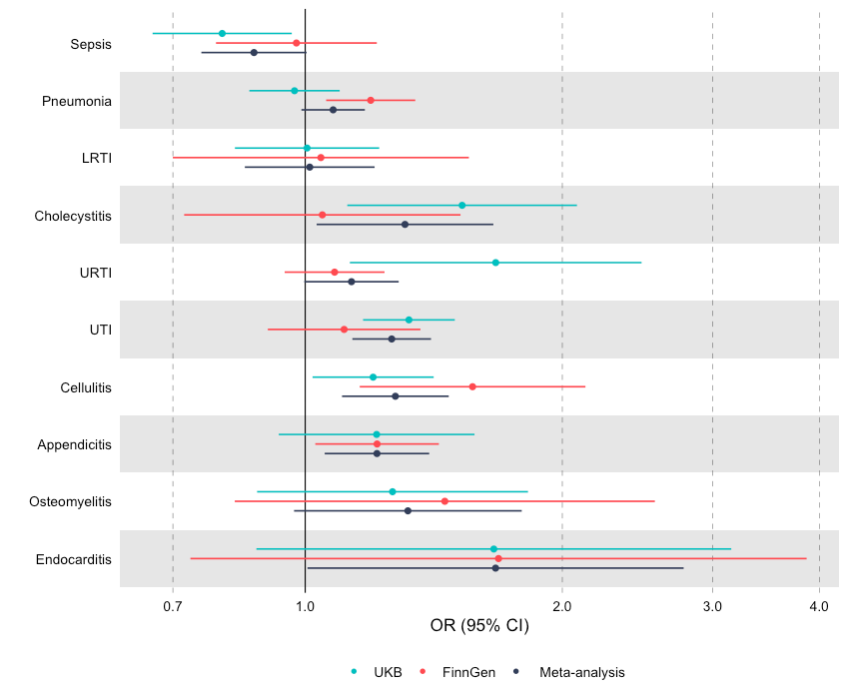

Supplement: S1 Fig — Results generated by IVW MR. (PNG) [file pmed.1004174.s002.png]

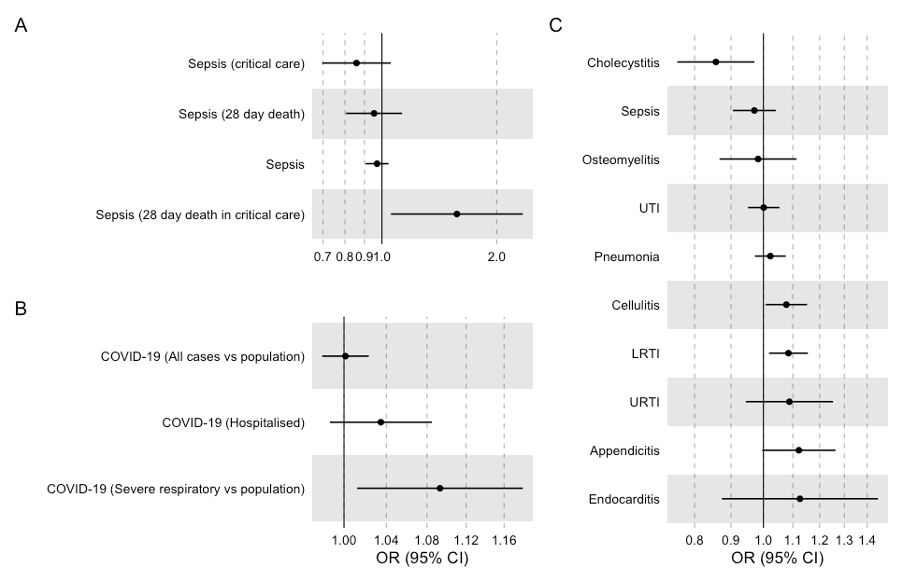

Supplement: S2 Fig — Effect estimates (odds ratios) generated by inverse variance weighted Mendelian randomisation for plasma protein levels of gp130 for (A) sepsis-related outcomes, (B) COVID-19-related outcomes, and (C) other UK Biobank–related outcomes. (PNG) [file pmed.1004174.s003.png]

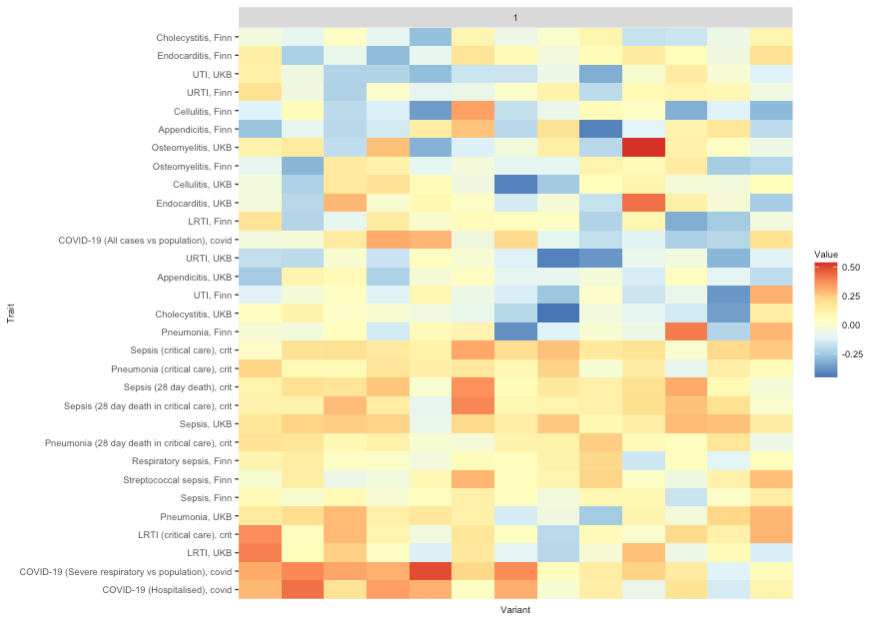

Supplement: S3 Fig — This includes 14 SNPs, with only one cluster identified. (PNG) [file pmed.1004174.s004.png]

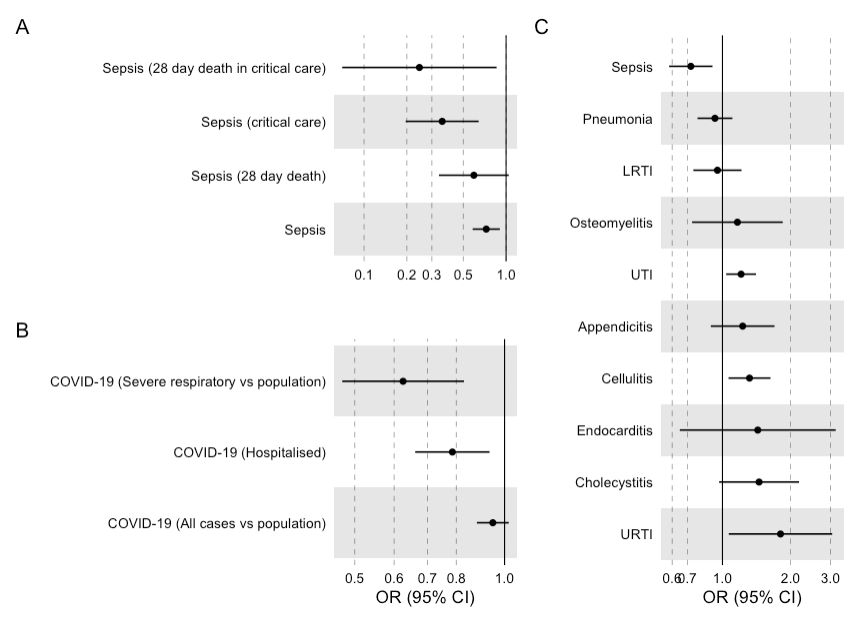

Supplement: S4 Fig — Effect estimates (odds ratios) generated by inverse variance weighted Mendelian randomisation for the 14 cisIL6R SNPs identified as a cluster by noise-augmented clustering for (A) sepsis-related outcomes, (B) COVID-19-related outcomes, and (C) other UK Biobank–related outcomes. (PNG) [file pmed.1004174.s005.png]

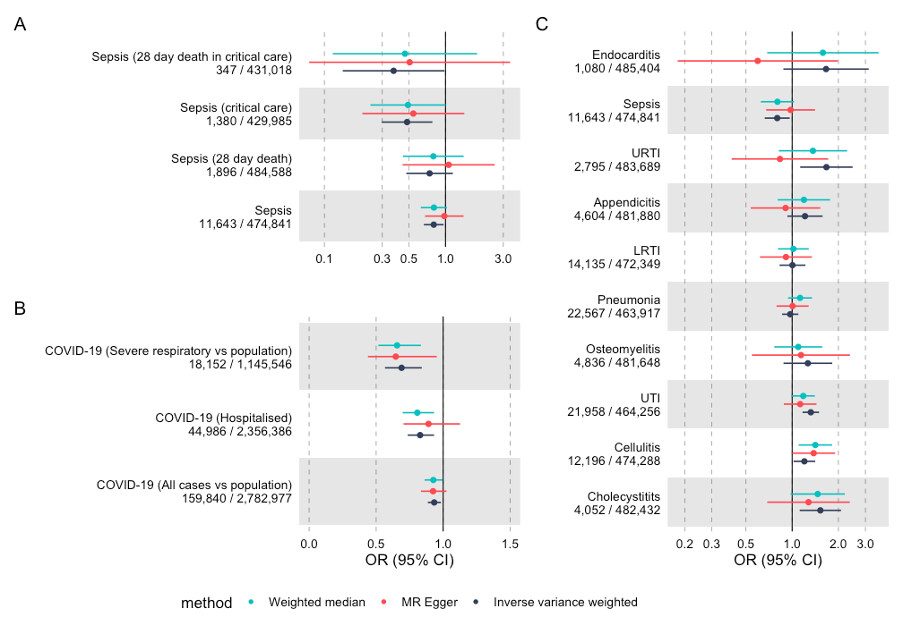

Supplement: S5 Fig — Effect estimates (odds ratios) generated by weighted median and MR-Egger meta-analytic approaches for (A) sepsis-related outcomes, (B) COVID-19-related outcomes, and (C) other UK Biobank–related outcomes. (PNG) [file pmed.1004174.s006.png]

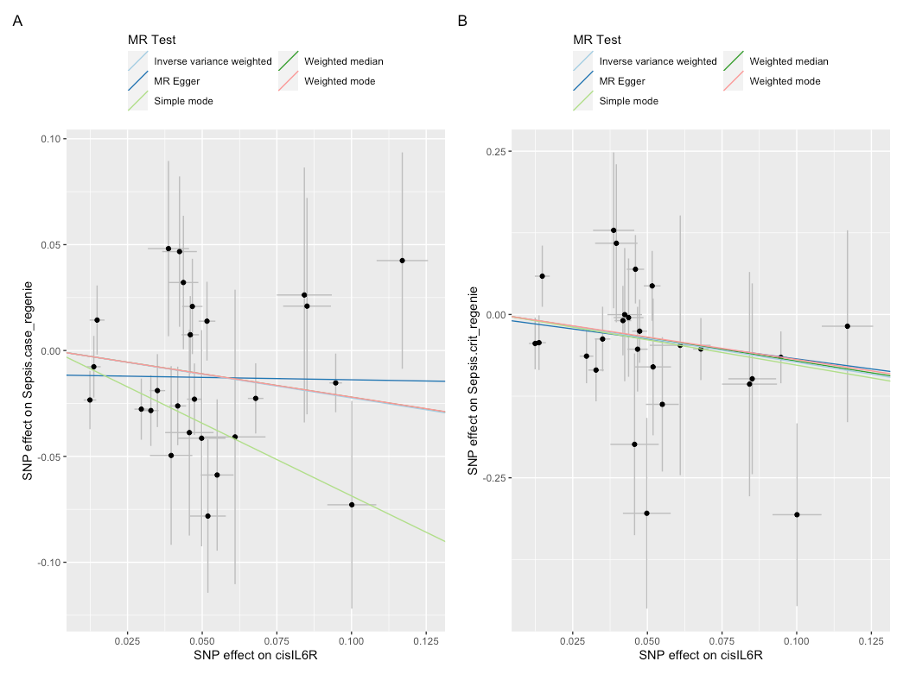

Supplement: S6 Fig — Each line represents the summarised effect estimate from a meta-analytic approach. (PNG) [file pmed.1004174.s007.png]

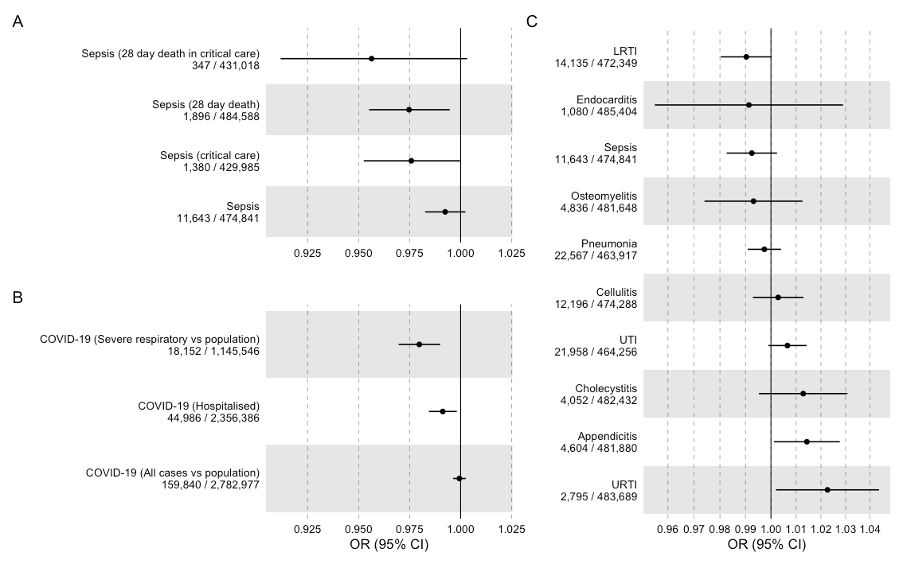

Supplement: S7 Fig — Odds ratios for sepsis generated by meta-analysing the SNP-outcome association (i.e., unweighted analysis) for 26 cisIL6R variants using inverse variance weighting and the effect on (A) sepsis-related outcomes, (B) COVID-19-related outcomes, and (C) other UK Biobank infection outcomes. (PNG) [file pmed.1004174.s008.png]

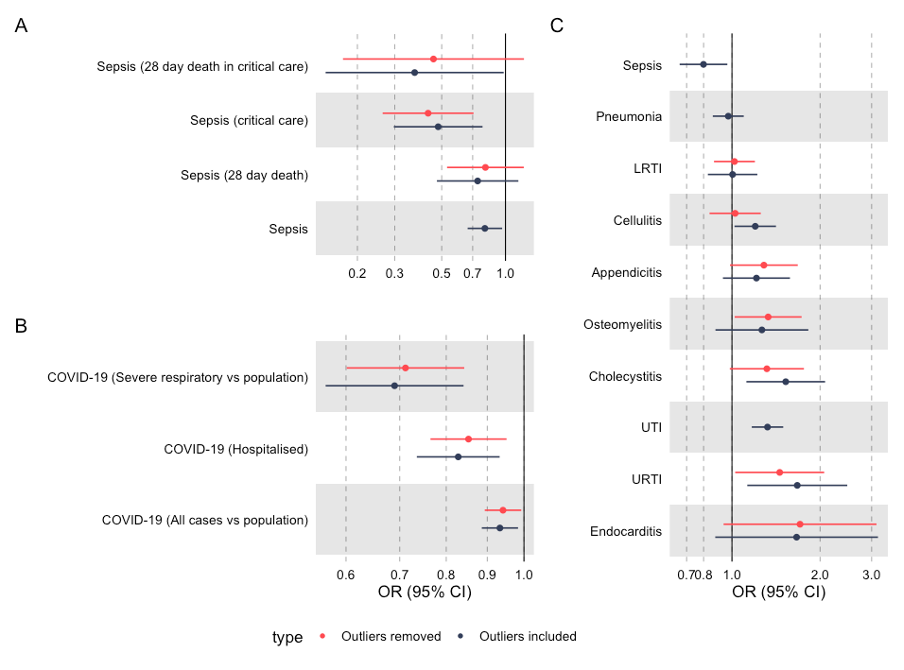

Supplement: S8 Fig — Results generated by IVW MR. Red represents the MR estimate with outliers removed, black the primary MR estimates. (PNG) [file pmed.1004174.s009.png]

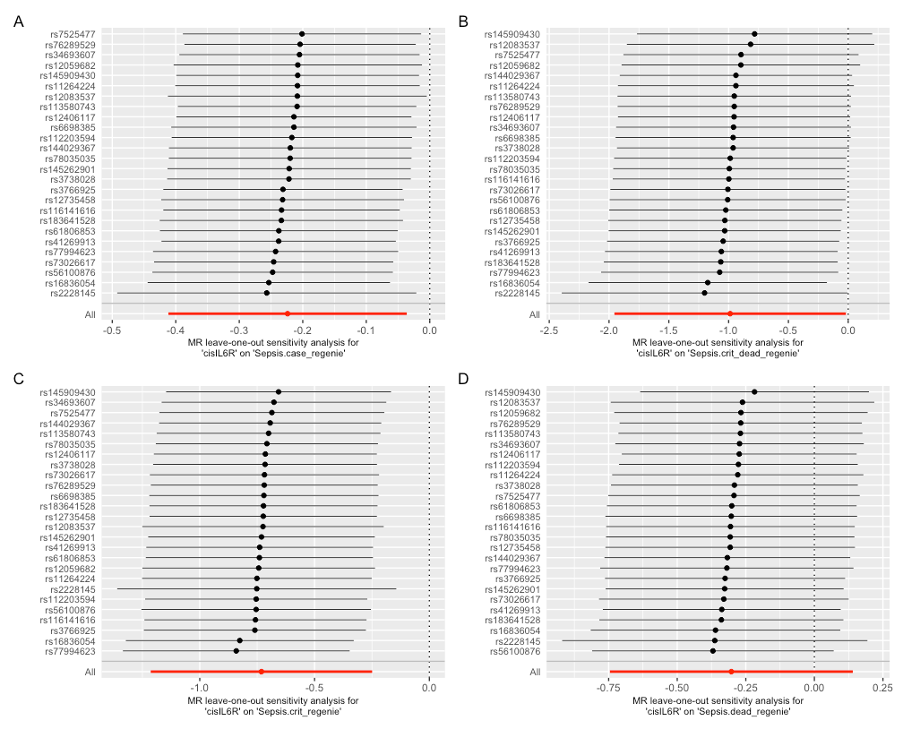

Supplement: S9 Fig — Inverse-variance weighted MR effect estimates (betas) when leaving out one SNP iteratively (leave-one-out analyses) for (A) critically unwell sepsis cases, (B) level 3 sepsis cases, (C) sepsis cases, and (D) sepsis-related mortality. (PNG) [file pmed.1004174.s010.png]

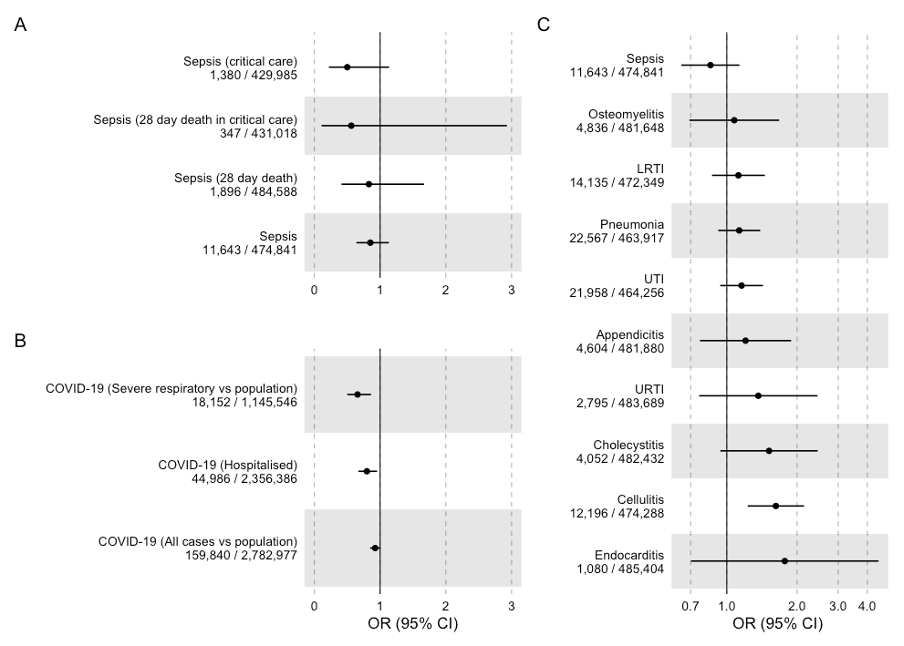

Supplement: S10 Fig — Odds ratios generated by the Wald ratio for the Asp358Ala (rs2228145) SNP association with (A) sepsis-related outcomes, (B) COVID-19-related outcomes, and (C) all other UK Biobank outcomes. (PNG) [file pmed.1004174.s011.png]
