# Supplementary material for: Therapeutic potential of IL6R blockade for the treatment of sepsis and sepsis-related death: A Mendelian randomisation study
Source: PLoS Med. 2023 Jan 30;20(1):e1004174. doi: 10.1371/journal.pmed.1004174 (PMC9925069; doi:10.1371/journal.pmed.1004174)
Supplement: Attachment — Submitted filename: Response letter_FINAL.docx [file pmed.1004174.s028.docx]

Dear Dr Davidson,

**Therapeutic potential of IL6R blockade for the treatment of sepsis and sepsis-related death: Findings from a Mendelian randomisation study**

**PMEDICINE-D-22-02409R1**

Many thanks for the time taken to edit and review our manuscript. We welcome the opportunity to provide a response to yourself and reviewers below. We have italicised and numerated the reviewers comments below, and provide our response on the line below, starting with “Response:”

We thank the reviewers for recognising the merits of our work and providing us an opportunity to improve our work.

Fergus Hamilton

On behalf of the authors

Editorial comments:

1. *… the authors sometimes over-interpret their findings – for example, in the Abstract they report a protective effect of IL6R blockade on “sepsis survival in critical care (OR=0.22; 95% CI 0.04-1.31)” ignoring the fact that the 95%CI is compatible with an opposite effect of increased risk of about 30%.*

Response: We understand that – at times – we may have given the impression of over-interpreting findings and have altered the text throughout the manuscript to reflect the imprecision in some of our estimates and to be more conservative in our position.

In abstract:

Line 48: addition of “although this result had a large degree of imprecision”

Line 52: Addition of “These results are subject to the limitations and assumptions of Mendelian randomisation, which in this case reflects interpretation of these SNP effects as causally acting through blockade of IL6R, and reflect lifetime exposure to IL6R blockade, rather than the effect of therapeutic IL6R blockade.”

Line 63: Removal of:

“IL6R blockade is causally associated with reduced incidence of sepsis, sepsis related critical care admission, and sepsis related mortality.”

Replaced with:

“IL6R blockade is causally associated with reduced incidence of sepsis. Similar but imprecisely estimated results supported a causal effect also on sepsis related mortality and critical care admission with sepsis.”

In discussion:

Line 878: Addition of “However, estimates for some phenotypes (e.g. death from critical care sepsis) were necessarily imprecise given the small sample size, although estimates were all concordant in direction.”

2. *Please update your title to ‘Therapeutic potential of IL6R blockade for the treatment of sepsis and sepsis-related death: a Mendelian randomisation study’.*

Response: We have done this.

3. *Please add ‘GDS is a member of PLOS Medicine’s editorial board’ to your Competing Interests.*

Response: We have done this.

4. *Please structure your Abstract using the PLOS Medicine headings (Background, Methods and Findings, Conclusions).*

Response: We have done this.

5. *Please ensure that all numbers presented in the abstract are present and identical to numbers presented in the main manuscript text.*

Response: We have done this.

6. *Please define the primary outcomes in your Abstract Methods and Findings.*

Response: We have edited the abstract to include a definition of key outcomes:

Line 33:

“We performed a Mendelian randomisation analysis using single nucleotide polymorphisms (SNPs) in and near *IL6R* to evaluate the likely causal effects of IL6R blockade on sepsis (primary outcome), sepsis severity, other infections, and COVID-19 (secondary outcomes).”

7. *In the last sentence of the Abstract Methods and Findings section, please describe the main limitation(s) of the study's methodology.*

Response: We have added such a sentence:

Line 52

“These results are subject to the limitations and assumptions of Mendelian randomisation, which in this case reflects interpretation of these SNP effects as causally acting through blockade of IL6R, and reflect lifetime exposure to IL6R blockade, rather than the effect of therapeutic IL6R blockade.”

8. *At this stage, we ask that you include a short, non-technical Author Summary of your research to make findings accessible to a wide audience that includes both scientists and non-scientists. The Author Summary should immediately follow the Abstract in your revised manuscript. This text is subject to editorial change and should be distinct from the scientific abstract. Please see our author guidelines for more information:* [*https://journals.plos.org/plosmedicine/s/revising-your-manuscript#loc-author-summary*](https://journals.plos.org/plosmedicine/s/revising-your-manuscript#loc-author-summary)

Response: We have now included one of these in the manuscript:

Line 68:

“Author summary

Why was this study done?

- Inhibition of the cytokine interleukin-6 (IL-6) using drugs such as Tocilizumab which bind to the IL-6 receptor has been shown to reduce mortality in critically unwell patients with COVID-19
- It is currently unknown whether IL-6 inhibition might have similar benefits in other, severe infections, such as bacterial sepsis.
- Genetic analyses (Mendelian randomisation) have previously predicted the success of IL-6 inhibition in COVID-19 and other conditions.

What did the researchers find?

- In a large, UK cohort (N=485,825, including 11,643 with sepsis), genetic variation acting as a proxy (or natural experiment) for IL6R blockade was associated with a reduced odds of sepsis (Odds ratio 0.80; 95% CI 0.66-0.96) in Mendelian randomisation analyses.
- Effects were consistent in secondary cohorts and when using differing definitions of sepsis, with effect sizes generally larger in more severe phenotypes.
- The effect estimates on sepsis were similar in magnitude to those seen in severe COVID-19 (Odds ratio 0.69, 95% 0.57 - 0.84), where IL-6 blockade is currently recommended.

What do the findings mean?

- Within the limitations and assumptions of Mendelian randomisation, these findings support the consideration of IL-6 inhibition in randomised controlled trials in sepsis.
- More broadly, these findings support the potentially pathological role of IL-6 in severe infection.”

9. *Please include continuous line numbering throughout your manuscript.*

Response: This has now been done.

10. *Please update your citations throughout to be normal script and placed within square brackets preceding punctuation.*

Response: This has now been done.

11. *Thank you for including a STROBE-MR checklist. Please update the checklist to use section headings/paragraph numbers rather than page numbers (these are likely to change in the event of publication).*

Response: This has now been done.

12. *Please cite your STROBE-MR checklist as ‘S1 checklist’ or similar, to ensure correct hyperlinking in the event of publication.*

Response: This has now been done.

13. *Please confirm that the licensing terms associated with Biorender.com permit publication under CC-BY creative commons attribution (used by PLOS Medicine).*

Response: We attach a CC-BY publishing licence from Biorender.com for this figure with this submission.

14. *Please cite your Supplementary Methods as ‘S1 Text’ or similar (as described here:* [*https://journals.plos.org/plosmedicine/s/supporting-information)*](https://journals.plos.org/plosmedicine/s/supporting-information))*.*

Response: This has now been done.

15. *Please relocation the information under the ‘Data availability’ subheading in the Methods is also captured in the Data Availability Statement section of the submission form (in the event of publication, this information will be published as metadata).*

Response: This has now been done.

16. *Table 1 (and throughout): Please do not report P=0.000, report as P<0.001.*

Response: This has now been done throughout the main text and tables.

17. *Please temper statements such as ‘Previous literature has not identified’ in the Discussion by adding ‘to our knowledge’.*

Response: Many thanks for this comment. We have adjusted this (line 485).

18. *The ‘Funding’ and ‘Conflicts of interest’ sections can be removed from the main text as all relevant information should be captured in the responses to the submission form (which will be published as metadata in the event of acceptance).*

Response: We have done this.

19. *The Ethics section should be relocated to the Methods.*

Response: We have done this.

20. *The inclusion of [Internet] in your References is only required where a journal is online only (otherwise it should be removed and cited as if print, even if access was made online).*

Response: We have updated our citation style to match PLOS Medicine’s formatting approach, and have therefore removed the [Internet] section.

21. *When citing preprints, please include [preprint] preceding the date of citation. If the preprint is now published, please update the reference accordingly.*

Response: We have updated all references and now do not cite any preprints.

Reviewers’ comments:

*Reviewer #1:*

1. *Looking at the scatter-plots in Supplementary Figure S3 Figure, there seem to be several clusters of instruments, one of which is even risk increasing. Is there the possibility that the cis-IL6R SNPs act via different pathways? Have you tried to cluster the IVs (e.g. using Noise-augmented directional clustering doi.org/10.1371/journal.pgen.1009975) to see if there is any evidence that the cis-IL6R SNPs form different pathways?*

Response: Thank you for this comment and for flagging this method. Despite recent advances in “cis” MR methodology, we are not aware of work specifically performing clustering on cis variants, where the prior likelihood of SNPs having effects via differing biological pathways is lower than in studies where a whole genome approach to identifying SNPs is used (such as in the referenced paper). Further, the type of analysis suggested here is to some extent focused on the challenge of managing common polygenic contributions to complex health outcomes/intermediates and qualitatively different to the approach of targeting cis variants with *a priori* assumptions justifying their selection. We completely agree that the results here are interesting and the possibility of substructure within a clutch of IL6R variants could offer insight into differential biology.

As an additional analysis, we performed clustering using the approach in the referenced paper and only identified one cluster of 14 SNPs (Supplementary Table 8). We then used these 14 SNPs in MR alone. These were consistent with our primary results. For example, in our primary analysis, the summary MR effect odds ratio for sepsis was 0.80; 95% CI 0.66-0.96, p = 0.019 , while using the clustered SNP’s, the OR was 0.72; 95% CI 0.58-0.90, p = 0.004.

We have added additional text to the methods (line 267) and results (line 412), while including results for this analysis in the supplement (Supplementary Figure S3 and S4, Supplementary Table 9)

2. *It would be interesting to perform a look-up in MR-Base if the genetic variants making up the cisILR6 instrument are associated with any other phenotypes that may be on the causal pathway from genetic variant to outcome. Especially in case if there are different clusters (See Comment 1), it would be great to understand what may cause the heterogeneity seen in the scatter-plot.*

Response: Thank you for this comment. This analysis has actually been performed recently.[1] In that analysis, a genetic risk score composed of 7 variants at *IL6R* was used as an instrument in a phenome wide association study. In that study, they report that genetically proxied IL-6 downregulation is associated with reduced risk of a number of cardiometabolic traits (including type 2 diabetes), with increased risk of urinary tract and skin infections (in line with our study) and atopic dermatitis.

We have added to the discussion some text (line 534) about pleiotropy at this locus:

“Focusing on variants within or near the *IL6R* it is difficult to completely rule out pleiotropic effects (e.g. these SNPs acting to reduce risk of sepsis by another, unrelated mechanism). IL-6 is highly pleotropic, and variants at *IL6R* have been shown to have effects on a wide range of clinical and biochemical traits, with a reduction in the odds of a number of cardiometabolic traits with genetically proxied IL-6 downregulation.”

3. *The description of sensitivity analysis c) (Methods: "ran the analysis weighted entirely on the SNP-outcome association (e.g. unweighted by CRP)" Results: "Secondly, we ran completely unweighted analyses (Supplementary Table 9, Supplementary Figure 4), which simply meta-analysed the effect of each SNP-outcome association, unweighted by any downstream effect.") is unclear. Does this refer to weights in the inverse-variance weighting or to the beta-coefficients? Please clarify*

Response: We apologies for the confusion here. In this analysis, we extracted the SNP-outcome association for each of the 26 SNPs included in our primary instrument, and then meta-analysed these SNPs by inverse variance weighting of this effect (e.g. the weight of their SNP-outcome association). We have rephrased this in the methods (line 252):

“and ran the analysis weighted entirely on the beta-coefficients from the SNP-outcome association (e.g. unweighted by CRP), with these results then meta-analysed together by the inverse variance weighting of each SNP-outcome (beta coefficient) association.”

and results (line 438).

“Secondly, we ran completely unweighted analyses (**Supplementary Table 9**, **Supplementary Figure 4**), which simply meta-analysed the effect of each SNP-outcome association, using inverse-variance weighting of the beta-coefficients at each SNP”

4. *Please add Legends to all Figures in the Supplement.*

Response: Thank you for this comment. We have added detailed figure legends to all supplementary figures.  *Minor comments*

1. *Please harmonize your notation of technical terms, e.g the manuscript contains both "Two Sample Mendelian randomisation study" and "two sample Mendelian randomisation study".*

Response: We have done this.

2. *Please clarify which weighting scheme was used for IVW, first or second order weights*

Response: Thank you for this comment. We used first-order weights. We have clarified this in the manuscript (line 217)

3. *When describing the sensitivity analysis in Methods Section "Sensitivity analyses:", please stick with either text or a list containing of a) b) and c). What list does the "c) ran the analysis weighted entirely" refer to?*

Response: Thank you for this comment. This was kept over from a previous version of the manuscript and have deleted the “c)”

4. *Please introduce abbreviations like IVW and MR at first use and then stick with abbreviation throughout the text. Similarly, in the Figure and Table Legends please be consistent if you use abbreviations or not.*

Response: Thank you for this comment. We have identified the first usage of these terms and used the abbreviations since. We have elected to keep the full names for all figures and tables. Occasionally, we use the term “Mendelian randomisation as full”, to be absolutely clear (e.g. in the study design portion of the methods).

*Reviewer #2:*

1. *Sepsis is a poorly characterized clinical syndrome; how consistently is it defined across your study cohorts?*

Response: We agree that the definition of sepsis is difficult, despite efforts to harmonise it. In this study, we utilised ICD-10 coding for sepsis for our main analysis (UK Biobank) and in follow up analyses (FinnGen). Both of these are derived from discharge coding. However, two of our cohorts (GAiNS and GenOSept) are derived from clinical cohort studies performed in intensive care units. As there is no gold standard definition of sepsis, this represents the true heterogeneity present in this diagnosis. It is therefore highly likely that the definition of the phenotype differs between and across these studies. We have added to the discussion on this point (line 498-510).

“Firstly, we rely on diagnostic coding for infections for both FinnGen and UKB, although we utilise clinically diagnosed cases in GenOSept and GAiNS. As sepsis is heterogenous and poorly characterised, these likely reflect slightly different pathological processes. Supporting this, some estimates in our replication cohort varied with the definition of sepsis, with much larger effects in those with specific sepsis codes (e.g. streptococcal sepsis) than with the main code.”

And

“Importantly, despite these differing study designs and approaches, nearly all estimates converged towards a protective effect of IL-6 inhibition.”

Despite these caveats, we feel the consistency of effect despite this heterogeneity provides confidence in our results; and suggests the effect is not unique to each cohort.

2. *You speak in the first sentence of sepsis as being characterized by elevated cytokine levels.  This is simplistic and inaccurate, given that the majority of genese impacte in sepsis are down-regulated.  Please try to be more biologically precise in your descriptions.*

Response: Thank you for this comment. We agree. This is a simplistic overview and not accurate. We have deleted this and rephrased (Line 83)

It now reads:

“Sepsis is a complex physiological and metabolic response to infection, characterised by dysregulation of the immune response and organ dysfunction”

3. *Your hypothesis would suggest a similar role for variants in gp130: can you analyze this as a complementary perspective on youor work?*

Response: many thanks for this comment. We agree this would be interesting and have performed a complementary analysis using variants cis to gp130 (also known as IL6ST). We do note, however, that the prior evidence supporting any association is much weaker, with no robust evidence of phenotypic associations of variation in gp130 being relevant.

In this analysis, we identified no consistent effect of plasma protein levels of gp130 on sepsis outcomes, although we do note an increase in the odds of death from critical care pneumonia (Odds ratio, OR 1.58; 95% CI 1.14-2.19), and death from critical care sepsis (OR 1.57; 95% CI 1.06-2.34). These results are intriguing but there was little consistency of effect, with the odds ratio for sepsis itself being close to the null (OR 0.95; 95% CI 0.81-1.13).

This analysis is reported in the methods (line 236), results (line 399), and discussion (line 888) with full results in Supplementary Table 7 and Supplementary Figure S2

4. *Figure 1 is confusing:  why is a distinction between cis and trans signaling relevant to your hypothesis?*

Response: Thank you for this comment. IL-6 regulation and action is complex and the use of genetic variation to provide insight into the impact of differential IL-6 function exposures has been complicated by the various forms of this. In anticipation of the likely reader appreciation of this complexity, we have kept this part of the paper as previously specified – given the nature of IL6 related Mendelian randomisation analyses in particular. However, we are happy to remove this or replace this with a more simple figure if requested editorially.

5. *Your variously speak of 28 and 30 day survival throughout the paper; please clarify.*

Response: Apologies for this error. All death data relates to 28-day mortality. We have edited this throughout the text.

6. *Why do you differentiate lower respiratory tract infection and pneumonia: to clinicians these are the same entity, or at least there is significant overlap.*

Response: Thank you for this comment. Although we agree that there is overlap between LRTI and pneumonia, we respectfully disagree that these should be combined. Firstly, they represent different pathological processes (even if imperfectly coded). Secondly, they have differing associations, with recent data from a comparative UK source showing that they are qualitatively different.[2]

We therefore feel it is important to run these analyses separately, given that they likely represent different (although similar) pathological processes.

7. *The stamen (PDF p 16) that "Conceptually our genetic instrument is similar to the action of anti-IL6R monoclonal antibodies (e.g. tocilizumab) that lead to complete inhibition of IL6R signalling by blocking both IL6 classical and trans signaling" is a bit of an over-reach.  Monoclonal antibodies do not necessarily result in complete inhibition andthe genomic region you study is not limited to IL-6R.*

Response: Thank you for this comment, this is very helpful. We agree that this statement was too strong and have reworded (line 301).

“Conceptually our genetic instrument is similar to the action of anti-IL6R monoclonal antibodies (e.g. tocilizumab) that lead to inhibition of IL6R signalling by blocking both IL6 classical and trans signalling, although the effect of inhibition is much reduced with our genetic instrument compared to therapeutic IL-6 inhibition”

However, we do feel that our genetic instrument has strong conceptual similarities to IL-6 inhibition, with empiric data supporting variation at this locus with subsequent randomised trial data.[3–6]

Although we accept that some of our variants are not in *IL6R* (but are close to it), this is a commonly accepted practice for “cis” MR.[7–9] Notably, when we perform MR with just the “canonical” rs2228145 / Asp358Ala SNP, our results are similar, but with less precision (as would be expected given the reduced power).

8. *The effect for endocarditis seems odd: can you elaborate?*

Response: Thank you. There are relatively few (1,080) cases of endocarditis in UK Biobank and only 546 cases in FinnGen. This leads to significant imprecision of estimates, although meta-analysed summary estimates across both cohorts suggest IL6R blockade may increase the risk of endocarditis.

In our view, this is consistent with the complex role of IL-6 in response to host infection. In both trial and registry data of IL-6 inhibition, increases in skin and soft tissue infection (usually streptococcal) and similar infections were identified,[10] which match our data (e.g. increased risk of cellulitis with IL6R blockade). Given endocarditis is commonly caused by streptococci, we suggest this effect may reflect the same pathophysiology, although this is not the focus of our study.

9. *In Table 1, the effects in sepsis are modest; the confidence intervals are wide, and much less impressive that what is seen for COVID.*

Response: Thank you for this comment. While we acknowledge a reduction in precision with our sepsis estimates, we disagree that this is “less impressive”. In our summary estimates, the point estimates for sepsis (and related outcomes) are similar and/or larger than the corresponding COVID-19 outcomes. For example, the COVID-19 HGI definition of severe COVID-19 (essentially requiring respiratory support) is most similar to our definition of critical sepsis (requiring intensive care support). The odds ratio for the former is 0.69 (95% CI 0.57-0.84, p = 0.0004), while the odds ratio for the latter is 0.48 (95% CI 0.3-0.78, p = 0.003).

What does differ between the estimates is the precision; which is driven by the vastly increased sample size for the severe COVID-19 outcomes (n case = 18,152 vs n case = 1,380). To demonstrate this, we re-ran analyses using our cis IL6R instrument on the COVID-19 HGI round 4, which only includes 3,886 cases (OpenGWAS ID: ebi-a-GCST010783), rather than round 7. In that analysis, the summary odds ratio was similar to the round 7 analyses (OR 0.56; 95% CI 0.39-0.81), but the p value was a log fold reduced (p = 0.002), and was actually smaller than the critical sepsis outcome, despite having four times as many cases.

Although it is challenging to directly compare these estimates, the magnitude of the effect in sepsis is similar if not larger to that in COVID-19, with the degree of precision that we would expect given the limited number of cases available for analyses.

Finally, the COVID-19 estimate reflects meta-analysis of a large number of studies with very similar study designs. We are unable to meta-analyse our estimates from our UK Biobank analyses with e.g. the data from GAiNS and GenOSept due to differing study designs, but the concordance of effect estimates despite these differences in study designs adds weight, in our view.

In line with editorial comments, we have softened our wording in the results to reflect some of this uncertainty (see response to Editor comment 1 for changes).

10. *The use of CRP as a marker of IL-6 bioactivity is potentially complicated because it is an acute phase reactant, identified through its role in pneumococcal infection, and so potentially impacted by other biologic influences.*

Response: Thank you for this comment. Instrumenting the IL-6 axis appropriately is difficult, and all approaches have limitations. We chose to use CRP for numerous reasons, which are justified in the supplementary methods, but recognise that this is not perfect, although it is commonly used.[1,11,12]

The availability of large scale GWAS of CRP allow us to generate multiple independent instruments (e.g. 26 cis IL6R variants) that significantly increase the power of this study. However, we do recognise this is imperfect. It is reassuring, therefore, that in an analysis which simply meta-analyses the SNP-outcome association at each of these 26 SNPs (Supplementary Figure S7, Supplementary Table 12) has similar results, suggesting that the weighting on CRP is not driving results.

11. *There are a number of grammatical errors or awkward formulations throughout.  A personal peeve is the use of the word "interrogate" to describe a scientific experiment.  One interrogates people, and may use inappropriate methods of suasion to gain a response; science tests hypotheses, and hopefully minimizes the biases associated with this.*

Response: Thank you for this comment. We have changed the word interrogate throughout the manuscript and have copy edited the manuscript throughout to improve readability.

*Reviewer #3:*

*…might be a good idea to discuss the rs2228145 SNP to deepen readers' understanding of the genetic marker.*

Response: Thank you for this comment. We have added some wording on this “canonical” SNP in the introduction, although we have kept this brief as this is not the focus of this manuscript:

Line 122:

“The same *IL6R* variants have been used in Mendelian randomisation (MR) studies as instruments for alterations in IL6R function and signalling, thus providing a functional proxy of IL6RA therapy, with an extensive literature relating to the canonical SNP, rs2228145. This SNP is known to lead to reduced levels of cell surface receptor that mediates classical signalling and a coupled increase in soluble IL6R that alters the setpoint for downstream IL-6 trans-signalling (**Figure 1**). A previous MR analysis suggested that reduced IL-6 signalling (modelled using rs2228145 alone) could reduce inflammation and risk of coronary heart disease, leading to clinical trials of monoclonal antibodies targeting either IL6R or IL-6 for coronary disease prevention. ”

*Reviewer #4:*

*It may constitute a valuable aspect to include the recently published work by Zhang et al. 2022	(doi: 10.3389/fimmu.2022.860703) in the discussion. Finally, this may lead to an improved	concept for clinical studies*

Response: Thank you for your kind review. The complexity of IL-6 biology is well recognised, and we thank the authors for the excellent reference which shows the pleiotropy of this locus, and also shows the relative paucity of studies on infection related traits despite this being a key immune locus. We have referenced it at line 539.

References:

1.	Georgakis MK, Malik R, Li X, Gill D, Levin MG, Vy HMT, et al. Genetically Downregulated Interleukin-6 Signaling Is Associated With a Favorable Cardiometabolic Profile: A Phenome-Wide Association Study. Circulation. 2021;143: 1177–1180. doi:10.1161/CIRCULATIONAHA.120.052604

2.	Millett ERC, Quint JK, Smeeth L, Daniel RM, Thomas SL. Incidence of community-acquired lower respiratory tract infections and pneumonia among older adults in the United Kingdom: a population-based study. Heimesaat MM, editor. PLoS One. 2013;8: e75131. doi:10.1371/journal.pone.0075131

3.	WHO Rapid Evidence Appraisal for COVID-19 Therapies (REACT) Working Group, Shankar-Hari M, Vale CL, Godolphin PJ, Fisher D, Higgins JPT, et al. Association Between Administration of IL-6 Antagonists and Mortality Among Patients Hospitalized for COVID-19: A Meta-analysis. JAMA. 2021;326: 499–518. doi:10.1001/jama.2021.11330

4.	Ridker PM, Devalaraja M, Baeres FMM, Engelmann MDM, Hovingh GK, Ivkovic M, et al. IL-6 inhibition with ziltivekimab in patients at high atherosclerotic risk (RESCUE): a double-blind, randomised, placebo-controlled, phase 2 trial. Lancet. 2021;397: 2060–2069. doi:10.1016/S0140-6736(21)00520-1

5.	Larsson SC, Burgess S, Gill D. Genetically proxied interleukin-6 receptor inhibition: opposing associations with COVID-19 and pneumonia. Eur Respir J. 2021;57. doi:10.1183/13993003.03545-2020

6.	Interleukin-6 Receptor Mendelian Randomisation Analysis (IL6R MR) Consortium, Swerdlow DI, Holmes MV, Kuchenbaecker KB, Engmann JEL, Shah T, et al. The interleukin-6 receptor as a target for prevention of coronary heart disease: a mendelian randomisation analysis. Lancet. 2012;379: 1214–1224. doi:10.1016/S0140-6736(12)60110-X

7.	Gill D, Burgess S. The evolution of mendelian randomization for investigating drug effects. PLoS Med. 2022;19: e1003898. doi:10.1371/journal.pmed.1003898

8.	Gill D, Georgakis MK, Walker VM, Schmidt AF, Gkatzionis A, Freitag DF, et al. Mendelian randomization for studying the effects of perturbing drug targets. Wellcome Open Res. 2021;6: 16. doi:10.12688/wellcomeopenres.16544.2

9.	Gkatzionis A, Burgess S, Newcombe PJ. Statistical methods for cis-Mendelian randomization with two-sample summary-level data. Genet Epidemiol. 2022. doi:10.1002/gepi.22506

10.	Singh JA, Wells GA, Christensen R, Tanjong Ghogomu E, Maxwell L, Macdonald JK, et al. Adverse effects of biologics: a network meta-analysis and Cochrane overview. Cochrane Database Syst Rev. 2011; CD008794. doi:10.1002/14651858.CD008794.pub2

11.	Georgakis MK, Malik R, Burgess S, Dichgans M. Additive Effects of Genetic Interleukin-6 Signaling Downregulation and Low-Density Lipoprotein Cholesterol Lowering on Cardiovascular Disease: A 2×2 Factorial Mendelian Randomization Analysis. J Am Heart Assoc. 2022;11: e023277. doi:10.1161/JAHA.121.023277

12.	Georgakis MK, Malik R, Richardson TG, Howson JMM, Anderson CD, Burgess S, et al. Associations of genetically predicted IL-6 signaling with cardiovascular disease risk across population subgroups. BMC Med. 2022;20: 245. doi:10.1186/s12916-022-02446-6
