# Supplementary material for: Therapeutic potential of IL6R blockade for the treatment of sepsis and sepsis-related death: A Mendelian randomisation study
Source: PLoS Med. 2023 Jan 30;20(1):e1004174. doi: 10.1371/journal.pmed.1004174 (PMC9925069; doi:10.1371/journal.pmed.1004174)
Supplement: Attachment — Submitted filename: Response letter_FINAL_R2.docx [file pmed.1004174.s029.docx]

Dear Dr Davidson,

**Therapeutic potential of IL6R blockade for the treatment of sepsis and sepsis-related death: Findings from a Mendelian randomisation study**

**PMEDICINE-D-22-02409R1**

Many thanks for accepting this manuscript. Our comments are below, after your editorial comments and the reviewer comments.

Fergus Hamilton

On behalf of the authors

Requests from Editors:

1. Data Availability Statement: ‘Access to the full summary statistics for the sepsis GWAS performed by the GaINS and GenOSept committee is by application to the relevant committee. This research was performed under UK Biobank application 56243. Individual access to UK Biobank can be arranged via the UK Biobank website.’ Please include relevant contact details (websites or email addresses) for data enquiries.

Response: This has been done.

2. Please include paragraph numbers as well as section headings in your STROBE-MR checklist.

Response: This has been done.

3. Please ensure citations precede punctuation throughout.

Response: This has been done.

4. The Funding, Competing Interests, and Data Availability sections can all be removed from the main text – this information is captured via the Submission Form and will be published as metadata alongside the article after acceptance.

Response: This has been done.

Comments from Reviewers:

1. Reviewer #1: Minor comments:

   The authors have added the following small detail to the methods and state that alternative meta-analytic approaches like MR-Egger and weighted median can test all three instrumental variable assumptions: 
   "We performed three broad types of sensitivity analysis. Firstly, we attempted to test the assumptions of MR (relevance, exclusion restriction, and indepenece) using alternative meta-analytic approaches (MR-Egger and weighted median approaches)."

   Importantly, MR-Egger and weighted median approaches only test for violations of the exclusion restriction assumption. They cannot verify that the exclusion restriction assumption holds, but only detect violations. Moreover, these methods do not test the relevance (to be tested with the F-statistic) or independence (untestable in summary-level setting) assumption. Please correct.

Response: This has been done.
